# Inter-zonal epithelial thickness differences for early keratoconus detection using optical coherence tomography

**DOI:** 10.1038/s41433-024-03199-7

**Published:** 2024-07-13

**Authors:** Tadas Naujokaitis, Ramin Khoramnia, Maximilian Friedrich, Hyeck-Soo Son, Gerd U. Auffarth, Victor A. Augustin

**Affiliations:** International Vision Correction Research Centre (IVCRC), Department of Ophthalmology, University of Heidelberg, Heidelberg, Germany

**Keywords:** Corneal diseases, Medical imaging

## Abstract

**Purpose:**

To develop and test a parameter for early keratoconus screening by quantifying the localized epithelial thickness differences in keratoconic eyes.

**Methods:**

The cross-sectional study included 189 eyes of 116 subjects in total: 86 eyes of 54 keratoconus patients with bilateral ectasia and 40 eyes of 20 healthy subjects in the parameter-development dataset and 42 eyes of 21 keratoconus patients with asymmetric ectasia and 21 eyes of 21 healthy subjects in the parameter-validation dataset. Epithelial thickness maps were obtained using anterior segment optical coherence tomography and the inter-zonal epithelial thickness differences were calculated. The developed parameter was tested in keratoconus patients with asymmetric ectasia.

**Results:**

Compared to healthy controls, the inferior-temporal and global inter-zonal epithelial thickness differences were higher not only in eyes with tomographically significant keratoconus (median [interquartile range] of 4.42 [3.13] µm vs. 0.78 [0.42] µm, *p* < 0.001, and 3.05 [1.51] µm vs. 1.07 [0.26] µm, *p* < 0.001, respectively), but also in tomographically normal keratoconus fellow eyes (1.36 [0.85] µm vs. 0.78 [0.42] µm, *p* = 0.005, and 1.31 [0.32] µm vs. 1.07 [0.26] µm, *p* = 0.01, respectively). The inferior-temporal inter-zonal epithelial thickness differences had an area under the receiver operating characteristic curve (95% confidence interval) of 0.991 (0.972–1) for detecting tomographically significant keratoconus and 0.749 (0.598–0.901) for differentiating between tomographically normal keratoconus fellow eyes and healthy controls.

**Conclusions:**

The inter-zonal epithelial thickness differences are increased in keratoconus fellow eyes which still have a normal Scheimpflug corneal tomography, and therefore may serve as a useful parameter to detect early ectatic changes.

## Introduction

Screening for subclinical ectasia in laser refractive surgery candidates is necessary to reduce the risk of postoperative ectasia [1–3]. Today, Scheimpflug corneal tomography has become a widely established method for ectasia detection, providing information on the anterior and posterior corneal curvature as well as the pachymetry [4]. While manifest ectasia can be identified readily, however, it still remains a challenge to detect signs of early ectasia [5].

Additional diagnostic modalities can help improve early keratoconus detection rate [6–10]. Corneal biomechanics demonstrated that weakening is found in many of the tomographically normal fellow eyes of keratoconus patients, confirming bilateral corneal pathology even in cases when keratoconus appears to be unilateral [6, 7]. Epithelial thickness (ET) mapping was also suggested to be helpful in detecting early ectasia [8, 9]. In today’s real-world clinical setting, the role of ET in keratoconus screening is often limited to visual assessment of ET maps and relies on the examiners’ experience. There is a need for simple and objective methods to utilize ET data in increasingly complex keratoconus diagnostics, and therefore this topic is being investigated by a growing number of studies.

A characteristic feature in keratoconus is localized epithelial thinning at the cone with a thickened epithelium around the cone, which results in high ET variation in the areas surrounding the cone [8]. We hypothesized that this locally increased variation in ET profile might help to detect eyes with early ectatic changes. In our study, we aimed to develop a parameter to quantify the localized ET differences in keratoconic eyes and to test its feasibility in screening for early ectasia in cases of eyes with normal Scheimpflug corneal tomography.

## Materials and methods

### Patients

This monocentric cross-sectional study was performed at the Department of Ophthalmology of the University of Heidelberg, Heidelberg, Germany, and consisted of two stages with two separate data analyses.

In the parameter-development stage, we included 86 eyes of 54 patients with keratoconus who showed bilateral ectasia on Scheimpflug corneal tomography, did not wear contact lenses for at least 14 days prior to examination, and had no history of corneal crosslinking or any other ocular surgery. In cases one eye did not fulfill the inclusion criteria due to history of crosslinking or other ocular surgery, only the fellow eye was included. Forty eyes of 20 healthy subjects with normal bilateral Scheimpflug corneal tomography, no history of ocular surgery, and no ocular pathology other than myopia, hyperopia, and astigmatism, served as a reference for normal ET.

In the parameter-validation stage, we included 21 keratoconus patients with very asymmetric ectasia, i.e. patients who showed tomographically-evident keratoconus in one eye and regular Scheimpflug corneal tomography in the fellow eye. Twenty-one eyes of additional 21 healthy subjects served as a control group. One eye of each healthy subject was randomly selected for inclusion in the study to provide a group comparable to keratoconus patients with asymmetric ectasia, each of whom had one seemingly-unaffected fellow eye.

The diagnosis of keratoconus was established after a complete clinical examination, including visual acuity testing, slit lamp examination, assessment of the clinical history, Scheimpflug corneal tomography (Pentacam AXL; Oculus Optikgeräte, Wetzlar, Germany) and corneal biomechanics (Corvis ST; Oculus Optikgeräte).

The study was approved by the Ethics Committee of the Medical Faculty of the University of Heidelberg (S-621/2021). All study procedures adhered to the tenets of the Declaration of Helsinki and written informed consent was obtained from all participants.

### Corneal tomography

Scheimpflug corneal tomography was performed using Pentacam AXL. We used Belin/Ambrósio Enhanced Ectasia total deviation index (BAD-D) as the main inclusion criterion on the presence of ectatic changes in corneal tomography. Although the BAD-D is not specific for keratoconus and therefore cannot be used alone to establish the diagnosis, it is commonly used for keratoconus-screening purposes as several studies found it to be one of the most sensitive Scheimpflug-tomographical indices for detection of corneal ectasia [11–13]. The corneal tomography was considered to represent a bilateral ectasia when the BAD-D was ≥2.69 in one eye and ≥1.65 in the other eye, which were the tomographic inclusion criteria for keratoconus patients with bilateral ectasia used in the parameter-development stage [14]. Keratoconus was defined as tomographically significant when the BAD-D was ≥2.69 [14]. Similar to the criteria used in previous studies, corneal tomography was defined as normal when the BAD-D was <1.65 and the tomographical parameters of Belin ABCD keratoconus classification had the stage 0 [5–7]. Therefore, the patients with very asymmetric ectasia were required to have the BAD-D of ≥2.69 in one eye and the BAD-D of <1.65 as well as the stage 0 of the Belin ABCD tomographic parameters in the fellow eye.

### Epithelial thickness measurement

ET maps using AS-OCT Anterion (Heidelberg Engineering, Heidelberg, Germany) were acquired with its integrated Cornea App. The device obtains B-scans with an axial resolution of approximately 8 µm using a light source of 1300 nm wavelength [15]. The ET maps are 7 mm in diameter and divided into 41 zones, which we labeled according to the illustration shown in Supplementary Fig. 1, and used average ET values of each zone for further analyses. We used one ET measurement per eye in the method development stage and three ET measurements per eye, performed on the same day, in the validation stage in order to evaluate measurement repeatability.

### Statistical analysis

We performed data analysis using the IBM SPSS Statistics Version 28 (International Business Machines Corporation, Armonk, NY, USA) and Microsoft Excel 365 (Microsoft Corporation, Redmond, WA, USA). As some ET data were not normally distributed according to the Shapiro-Wilk test, we used non-parametrical tests and provided medians with interquartile ranges (IQR). The performance of epithelial parameters to differentiate between healthy and keratoconus patients‘ eyes was assessed using the receiver operating characteristic (ROC) analysis, including the area under the curve (AUC), sensitivity and specificity values. The correlation of the developed epithelial parameters with the corneal tomographic parameters was evaluated using the Spearman correlation coefficient. The eyes of keratoconus patients were compared with healthy controls in the parameter-validation stage using the Mann–Whitney U test, with the two-sided significance level of 0.0125 after the Bonferroni’s correction for 4 comparisons. The repeatability the measured zonal ET and the calculated inter-zonal ET differences was assessed using the intra-subject standard deviation (SD) of the three repeated measurements.

### Parameter development

The mean absolute ET differences between each zone and its neighboring zones (inter-zonal ET differences) were calculated using the following formula:

$${{{{{\rm{inter}}}} {\mbox{-}}{{\rm{{zonal}}}}}}\; {{{{{\rm{ET}}}}}}\; {{{{{\rm{differences}}}}}}(a)=\frac{{\sum }_{i=1}^{n}|{{ET}}_{{zone}}\left(a\right)-{{ET}}_{{zone}}({x}_{i})|}{n}$$

where ***a*** is the zone for which the inter-zonal ET differences are calculated, ***n*** is the number of neighboring zones, ***x***_**1**_, ***x***_**2**_, …, ***x***_***n***_ are the neighboring zones, and ET_zone_ is the mean ET of a zone.

We performed ROC analysis to identify zones with the largest AUC in differentiating keratoconus from healthy eyes. In order to create a parameter describing localized ET differences in keratoconus, we selected two zones that showed the largest AUC values (the paracentral inferior zone with the AUC of 0.974 and the paracentral inferior-temporal zone with the AUC of 0.970), and calculated the average inter-zonal ET differences of these two zones for each eye:

$$	{{{{{\rm{inferior}}}}}}{\mbox{-}}{{{{{\rm{temporal}}}}}}\; {{{{{\rm{inter}}}}}}{\mbox{-}}{{{{{\rm{zonal}}}}}}\; {{{{{\rm{ET}}}}}}\; {{{{{\rm{differences}}}}}}\\ 	=\frac{{{{{\rm{inter}}}}}{\mbox{-}}{{{{\rm{zonal}}}} \; {{{\rm{ET}}}} \; {{{\rm{differences}}}}}\, \left({{{{{\rm{cI}}}}}}\right)+{{{{\rm{inter}}}}}{\mbox{-}}{{{{\rm{zonal}}}}\;{{{\rm{ET}}}}\;{{{\rm{differences}}}}}({{{{{\rm{cTI}}}}}})}{2}$$

where ***cI*** is the paracentral inferior zone and ***cTI*** is the paracentral inferior-temporal zone.

To describe global ET differences, we calculated the mean inter-zonal ET difference value of all 41 zones (global inter-zonal ET differences) in each eye using the following formula:

$${{{{{\rm{global}}}}}}\; {{{{{\rm{inter}}}}}}{\mbox{-}}{{{{{\rm{zonal}}}}}}\; {{{{{\rm{ET}}}}}}\; {{{{{\rm{differences}}}}}}=\frac{{\sum }_{{{{{{\rm{i}}}}}}=1}^{41}{{{{\rm{inter}}}}}{\mbox{-}}{{{{\rm{zonal}}}}\; {{{\rm{ ET}}}}\; {{{\rm{differences}}}}}({{{{{{\rm{x}}}}}}}_{{{{{{\rm{i}}}}}}})}{41}$$

where ***x***_***1***_, ***x***_***2***_, …, ***x***_***41***_ are the ET map zones.

According to the sensitivity and specificity values in the ROC analysis, we selected cut-off values for the developed parameters: a lower cut-off value with a sensitivity of at least 95% to detect early ET changes and a higher cut-off value with a specificity of at least 95% to minimize the false-positive rate.

Finally, to evaluate whether the developed parameters correlate with the corneal tomographic changes in keratoconus, we plotted the data and calculated the Spearman correlation coefficients for the BAD-D and the tomographic parameters of the Belin ABCD keratoconus classification.

### Parameter validation

Based on the data from the parameter-development dataset, 12 eyes per group were required to reach 80% power of detecting a difference in means in both the inferior-temporal and global inter-zonal ET differences at a two-sided local significance level of 0.0125, assuming normality for the purpose of sample size calculation. To protect against the loss of power from deviations from the distributional assumption, we increased the sample size in the parameter-validation stage of the study to 21 eyes per group.

We calculated the inter-zonal ET differences for each measurement in patients with very asymmetric ectasia and in healthy controls and used the mean values of the three measurements for further analysis. We also assessed the repeatability of the measured zonal ET and the calculated inter-zonal ET differences.

To evaluate whether the newly-developed parameters can be useful in detecting ectasia, their values in healthy controls were compared with those in tomographically normal eyes of very-asymmetric-ectasia patients as well as with eyes with tomographically significant keratoconus using Mann–Whitney U test. We also performed ROC analysis and evaluated the repeatability of the developed parameters.

## Results

### Parameter development

In the parameter-development dataset, the median (IQR) BAD-D value was 6.4 (4.8) in eyes with keratoconus and 1.0 (0.8) in healthy eyes (*p* < 0.001). The characteristics of patients and eyes are presented in Supplementary Table 1.

We observed epithelial thinning in the paracentral inferior-temporal area and a slight thickening in some of the surrounding zones in keratoconus eyes. In the inter-zonal ET difference maps, they had higher values in the inferior-temporal quadrant with median (IQR) values of 4.2 µm (3.6 µm) and 4.2 µm (2.8 µm) in the paracentral inferior and the paracentral inferior-temporal zones, respectively. Healthy eyes exhibited mostly uniformly-distributed low median inter-zonal ET difference values of approximately 1 µm (Supplementary Fig. 2). The inter-individual variability of the inter-zonal ET differences was considerably lower than of the zonal ET values in both healthy and keratoconus eyes (Supplementary Fig. 3).

**Fig. 1:**
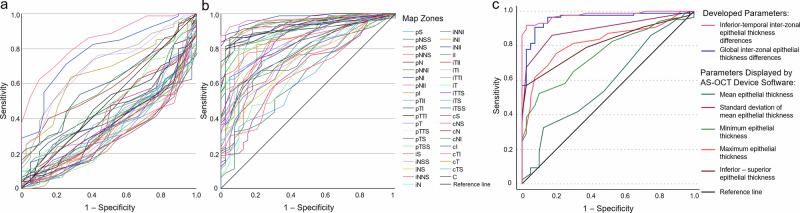
Receiver operating characteristic curves of the parameter-development dataset. Receiver operating characteristic curves for differentiating between keratoconus and healthy eyes using zonal epithelial thickness (**a**) and inter-zonal epithelial thickness difference (**b**) values, as well as current vs. developed epithelial parameters (**c**) in the parameter-development dataset. Lower zonal epithelial thickness values were considered to be indicative for keratoconus in the graph **a**.

ROC analysis revealed better performance of the inter-zonal ET differences in comparison to the zonal ET in differentiating keratoconus from healthy controls, with the highest AUC values in the inferior-temporal area (Fig. 1, Supplementary Table 2).

The localized parameter (inferior-temporal inter-zonal ET differences) had an ROC AUC of 0.978 (95% confidence interval [95%CI] of 0.957–0.999). Using a cut-off value of 1.21 µm, the parameter reached a sensitivity of 96.5% and a specificity of 82.5%. With a higher cut-off value of 1.75 µm, it had a sensitivity of 91.9% and a specificity of 97.5%. The ROC AUC of the global parameter (global inter-zonal ET differences) was 0.956 (95%CI 0.920–0.993). With a cut-off value of 1.42 µm, it had a sensitivity of 96.5% and a specificity of 85.0%. When using 1.88 µm as the cut-off, the parameter reached a sensitivity of 81.4% and a specificity of 95.0%.

Both the localized and the global inter-zonal ET difference parameters correlated statistically significantly with the BAD-D index and the tomographic parameters of the Belin ABCD keratoconus classification (*p* < 0.001 for all the analyzed parameters). The correlation was better for the global parameter, especially in more advanced keratoconus. The scatter plots and Spearman correlation coefficient values are presented in Supplementary Fig. 4.

### Parameter validation

The characteristics of patients and eyes in the parameter-validation dataset are presented in Supplementary Table 1. The tomographically normal fellow eyes of keratoconus patients did not differ statistically significantly from healthy controls in terms of Scheimpflug-tomographical parameters, including the Db value of the Belin/Ambrósio Enhanced Ectasia display, which indicates the deviation from the normative database of the posterior elevation. The median (IQR) Db value was −0.1 (0.7) in the tomographically normal fellow eyes and −0.1 (0.9) in healthy controls (*p* = 0.589, Mann–Whitney U test).

**Fig. 2:**
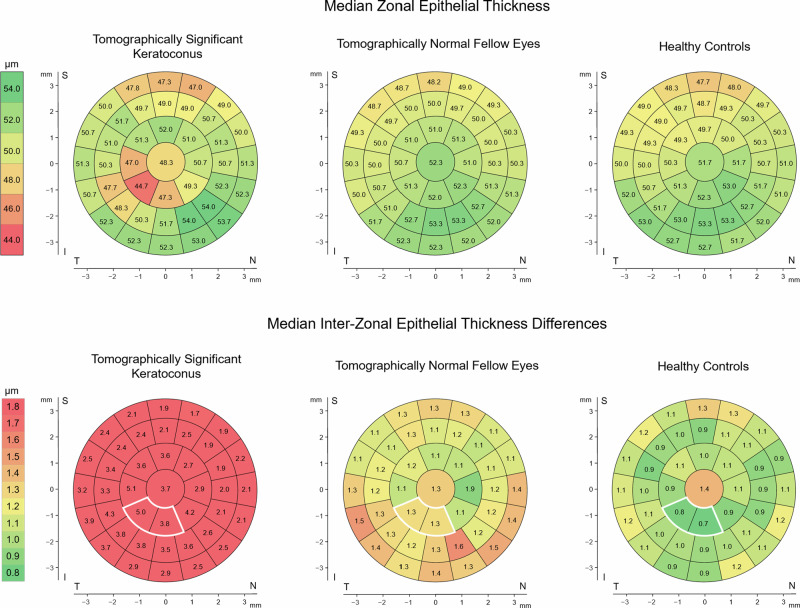
Median zonal epithelial thickness and median inter-zonal epithelial thickness differences of the eyes included in the parameter-validation dataset. The white border indicates the values used to calculate the inferior-temporal inter-zonal epithelial thickness differences. All values in the maps are in µm. I inferior, N nasal, S superior, T temporal.

Figure 2 shows the median zonal ET and the inter-zonal ET differences of the eyes in the parameter-validation dataset. The tomographically normal fellow eyes tended to have higher median inter-zonal ET differences compared to healthy eyes, especially in the inferior-temporal area. The median intra-individual SD of three repeated measurements, displayed in Supplementary Fig. 5, was 0.6 µm for the zonal ET and up to 0.3 µm for the inter-zonal ET differences in most zones in both keratoconus patients and healthy controls.

**Fig. 3:**
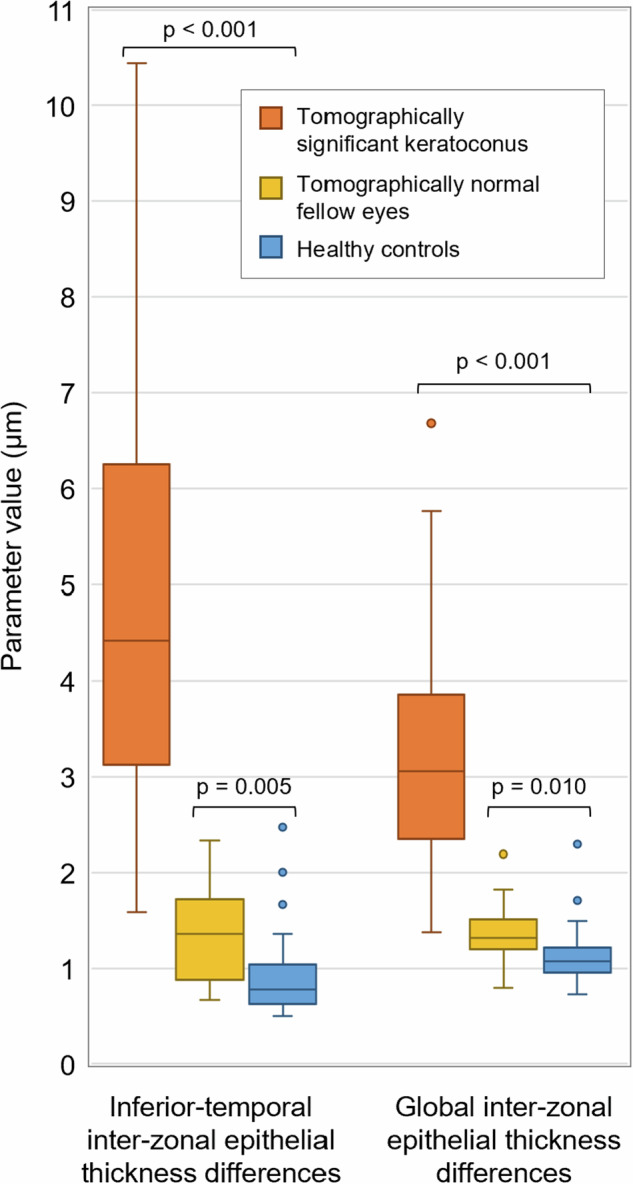
Box plots of inferior-temporal and global inter-zonal epithelial thickness differences in tomographically significant keratoconus, tomographically normal keratoconus fellow eyes, and healthy controls. Mann–Whitney U test was used to compare the eyes of keratoconus patients with healthy controls (two-sided significance level of 0.0125 after the Bonferroni’s correction for 4 comparisons).

The median (IQR) inferior-temporal inter-zonal ET differences were 4.42 µm (3.13 µm) in eyes with tomographically significant keratoconus, 1.36 µm (0.85 µm) in tomographically normal fellow eyes of keratoconus patients, and 0.78 µm (0.42 µm) in healthy controls. The median (IQR) global inter-zonal ET differences were 3.05 µm (1.51 µm), 1.31 µm (0.32 µm), and 1.07 µm (0.26 µm), respectively. Compared to healthy controls, both the inferior-temporal and global inter-zonal ET difference parameters were statistically significantly higher not only in eyes with tomographically significant keratoconus (*p* < 0.001 for both parameters), but also in tomographically normal fellow eyes of keratoconus patients (*p* = 0.005 and *p* = 0.010, respectively; Fig. 3). The median (IQR) intra-individual SD of three repeated measurements was 0.25 µm (0.14 µm) and 0.10 µm (0.07 µm) in tomographically significant keratoconus, 0.20 µm (0.13 µm) and 0.07 µm (0.09 µm) in tomographically normal fellow eyes, 0.12 µm (0.12 µm) and 0.12 µm (0.09 µm) in healthy controls for the inferior-temporal and the global inter-zonal ET differences, respectively.

**Fig. 4:**
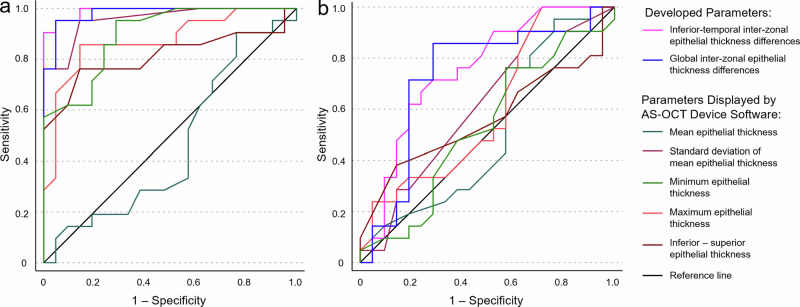
Receiver operating characteristic curves of the parameter-validation dataset. Receiver operating characteristic curves for differentiating between tomographically significant keratoconus and healthy eyes (**a**), and between tomographically normal keratoconus fellow eyes and healthy eyes (**b**) in the parameter-validation dataset. Area under the curve (95% confidence interval) values were 0.991 (0.972–1) and 0.749 (0.598–0.901) for inferior-temporal inter-zonal epithelial thickness differences, 0.982 (0.951–1) and 0.732 (0.567–0.898) for global inter-zonal epithelial thickness differences, 0.465 (0.285–0.645) and 0.515 (0.333–0.697) for mean epithelial thickness, 0.958 (0.904–1) and 0.611 (0.439–0.784) for standard deviation of mean epithelial thickness, 0.898 (0.808–0.988) and 0.531 (0.352–0.709) for minimum epithelial thickness, 0.874 (0.764–0.984) and 0.600 (0.424–0.775) for maximum epithelial thickness, 0.814 (0.673–0.955) and 0.563 (0.384–0.743) for inferior–superior epithelial thickness, when detecting tomographically significant keratoconus and tomographically normal fellow eyes, respectively.

**Fig. 5:**
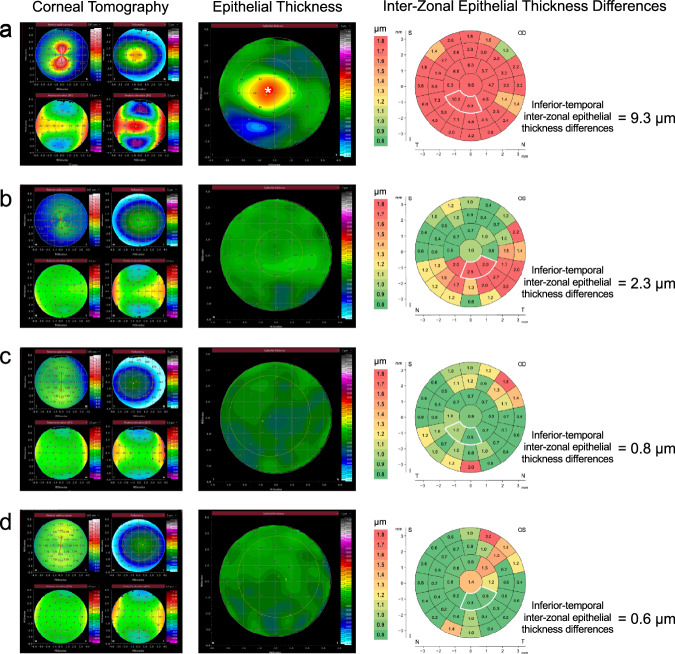
Examples of corneal tomography, epithelial thickness, and inter-zonal epithelial thickness differences in keratoconus and healthy eyes. Representative examples of corneal tomography, epithelial thickness, and inter-zonal epithelial thickness difference maps in tomographically significant keratoconus (**a**), tomographically normal keratoconus fellow eye with epithelium showing early ectatic changes (**b**), tomographically normal keratoconus fellow eye with a normal epithelial thickness profile (**c**), and healthy eye (**d**). The inferior-temporal inter-zonal epithelial thickness differences, shown on the right side, are based on the values marked with the white border in the inter-zonal epithelial thickness difference maps. The parameter excludes the peripheral zones, which frequently had elevated inter-zonal epithelial thickness difference values in healthy eyes.

Using the cut-off value of 1.21 µm, derived from the parameter-development dataset, the inferior-temporal inter-zonal ET differences could differentiate tomographically normal keratoconus fellow eyes from healthy controls with 57.1% sensitivity and 81.0% specificity. The cut-off value of 1.75 µm resulted in a sensitivity of 19.0% and a specificity of 90.5%. The global inter-zonal ET difference parameter reached 38.1% sensitivity and 81.0% specificity when using the cut-off value of 1.42 µm. However, it had a sensitivity of only 4.8% when using the higher cut-off value of 1.88 µm (specificity of 95.2%). The localized inferior-temporal inter-zonal ET difference parameter had an AUC of 0.749 (95%CI of 0.598–0.901) and the global inter-zonal ET difference parameter had an AUC of 0.732 (95%CI of 0.567–0.898) (Fig. 4). In case of tomographically significant keratoconus, the sensitivity values were 100% and 95.2% for the inferior-temporal inter-zonal ET differences, and 95.2% and 90.5% for the global inter-zonal ET differences, with the lower and the higher cut-off values, respectively. Examples of individual AS-OCT-based corneal tomography, ET, and inter-zonal ET difference maps are shown in Fig. 5.

## Discussion

The ET maps were first obtained using high-frequency ultrasound in 1994 [16]. It was shown that the epithelium of the normal cornea is thinner superiorly and temporally by 5.9 µm and 1.3 µm at the 3-mm radius, respectively, probably due to the eyelid anatomy [17]. Apart from this, the differences in the ET of healthy eyes are relatively low and the epithelial profile is smooth [17]. The ET maps of healthy eyes in our study shared similar characteristics. Studies on epithelial remodeling revealed the function of the epithelium to regularize the corneal surface by thickening above stromal depressions and thinning above stromal elevations [18, 19]. It also occurs in keratoconus, where the epithelium becomes thinner above the protruding stromal cone and thicker around it [19, 20]. The epithelium masks early keratoconic changes in the anterior corneal surface, and an ET analysis can therefore be useful to detect early ectasia [19].

In contrast to the ultrasound-based ET measurements, the AS-OCT-based ET mapping is a non-contact measurement and the acquisition time is considerably shorter [15, 21]. A study comparing the ET maps obtained using very-high-frequency ultrasound and spectral-domain AS-OCT found that the ET measurements obtained with both systems were highly correlated, although not interchangeable [22]. The AS-OCT was shown to provide highly-repeatable ET measurements both in normal and keratoconus eyes [15, 23]. Feng et al. compared the ET measurements performed using Anterion AS-OCT, the device used in our study, and Avanti (Optovue, Inc, Fremont, CA, USA) AS-OCT [15]. Although the Anterion device uses a longer wavelength than Avanti (1300 nm vs. 840 nm), which is disadvantageous for the axial resolution of a scan (approximately 8 µm vs. 5 µm), the study reported the measurement repeatability of Anterion to be better than that of Avanti [15]. The authors assumed this could be due to a higher number of the radial scans obtained with Anterion (65 vs. 8 scans) and its eye-tracking ability [15].

While a number of existing parameters can describe the ET profile, they have considerable limitations when used in ectasia diagnostics. The mean ET is not suitable for this purpose as we found it to be similar in healthy and keratoconus eyes. The mean zonal ET differs between healthy and keratoconus eyes in some zones [24] but its high inter-individual variation in healthy eyes limits its use for keratoconus detection. Therefore, it may be beneficial to disregard the absolute ET values and analyze the differences within a particular ET map only. One approach is to calculate the difference between the maximum and minimum ET [25]. These values, however, may not be ideal in early ectasia diagnostics, as we observed the minimum ET value to refer to the naturally-occurring thinner superior peripheral epithelium instead of the beginning inferior-temporal epithelial thinning in keratoconus in multiple occasions. Another approach is to use global parameters describing ET differences, such as the SD of the mean ET and the ET pattern deviation [9, 21, 26–28]. Still, they are influenced by ET changes in areas less relevant in keratoconus diagnostics, such as the elevated ET variation in peripheral zones, which we observed in healthy eyes. As the epithelial thinning in keratoconus usually occurs in the inferior-temporal area, some authors suggested analyzing this area only [9, 27, 29]. For example, Pavlatos et al. developed a coincident thinning index based on the Gaussian fits of ET pattern deviation maps in an inferiorly-temporally-centered search area [29]. Their approach requires normative ET data to calculate ET pattern deviation, as well as custom algorithms for the segmentation of the AS-OCT images and the calculation of the index, and therefore can only be used in a research setting at present [29]. Other approaches to utilize ET data in keratoconus diagnostics include step-wise algorithms with a manual ET map inspection, utilizing machine learning to combine multiple tomographic and ET parameters, and using custom-build ultrahigh-resolution AS-OCT devices [9, 26, 27, 30–32].

In our study, we sought to develop a new parameter that quantifies localized ET variation in keratoconus. It uses zonal values which are displayed by the AS-OCT-device software and therefore there is no need to access raw data. The developed inter-zonal ET difference parameter describes how much the mean ET of the analyzed zone differs from the values of the surrounding zones. It can be graphically displayed as a color-coded map to enable a quick overview of an individual ET profile variation. We observed low inter-individual variation of the inter-zonal ET differences in healthy eyes and high repeatability in both healthy and keratoconus eyes.

Although the inter-zonal ET differences detected early ectatic changes in more than half of the eyes which were shown to be tomographically normal on Scheimpflug corneal tomography, their specificity is insufficient to diagnose keratoconus based on their values alone. This may be because the inter-zonal ET differences could also be elevated due to various other factors which cause irregular ET profile, such as dry eye, contact lens wear, or corneal scarring. Instead, the inter-zonal ET differences could be used to screen for eyes with an elevated ET variation, which necessitate a careful ET map examination to exclude the presence of ectasia. In addition, in some eyes with a predisposition to ectasia but no tomographic ectatic changes, the ET profile may still be normal as at least a slight stromal ectatic change is necessary for the epithelial remodeling to occur. In general, all epithelial parameters should be evaluated together with tomographic and biomechanical data. It remains the subject of future studies to investigate whether another method of calculating the inter-zonal ET differences and the integration of them into ectasia-detection algorithms can further improve early keratoconus detection.

The keratoconus patient population in our study was male-predominant, especially in asymmetric keratoconus, which is similar to the distribution reported by Vinciguerra et al. [6]. This highlights the need of studies investigating risk factors of developing asymmetric ectasia, as it appears to be more common in males [6, 7].

A limitation of the study is the relatively small sample size of patients with an asymmetric ectasia, which is ascribable to the rarity of such cases [5, 7]. We chose to examine this patient population as they provide insights into the earliest ectatic changes before they can be detected on Scheimpflug corneal tomography. The inclusion criteria used were more stringent than in most other studies on epithelial parameters, which resulted in lower sensitivity values in our study [9, 27, 29]. In addition, some of the tomographically normal eyes without elevated inter-zonal ET differences could actually be true negatives [8]. We demonstrated, however, that the inferior-temporal inter-zonal ET differences were elevated in more than half of the eyes which were tomographically normal, indicating the additional value of this epithelial parameter in ectasia detection.

Currently, the epithelial parameters are not used to evaluate keratoconus progression [33]. As we could demonstrate a correlation between the inter-zonal ET differences and the tomographical changes in keratoconus, future studies should investigate whether the inter-zonal ET differences can be useful as an additional parameter when assessing keratoconus progression.

In conclusion, we found the inter-zonal ET differences to correlate with the tomographical changes in keratoconus and to be increased not only in tomographically significant keratoconus, but also in tomographically normal keratoconus fellow eyes compared to healthy eyes. Therefore, the inter-zonal ET differences may present a novel means to detect early ectasia.

Supplemental material is available at Eye’s website.

## Summary

### What was known before

- Scheimpflug corneal tomography is an established method in keratoconus diagnostics but early ectasia detection remains challenging. The epithelium masks early keratoconic changes in the anterior corneal surface.

### What this study adds

- The inter-zonal epithelial thickness differences are increased not only in tomographically significant keratoconus, but also in tomographically normal keratoconus fellow eyes. The developed parameter could be used to screen for eyes with early ectatic changes before they can be detected with Scheimpflug corneal tomography.

## Supplementary information

Supplementary Table 1

Supplementary Table 2

Supplementary Figure 1

Supplementary Figure 2

Supplementary Figure 3

Supplementary Figure 4

Supplementary Figure 5

Supplementary materials

## Data Availability

Data are available from the corresponding author on reasonable request.
