# Supplementary material for: Inter-zonal epithelial thickness differences for early keratoconus detection using optical coherence tomography
Source: Eye (Lond). 2024 Jul 13;38(15):2968–75. doi: 10.1038/s41433-024-03199-7 (PMC11461491; doi:10.1038/s41433-024-03199-7)
Supplement: Supplementary file 1 — Supplementary Table 1 [file 41433_2024_3199_MOESM1_ESM.docx]

**Supplementary Table 1.** Characteristics of patients and eyes included in the study.

| **Characteristic** | **Parameter-development dataset** | | | **Parameter-validation dataset** | | | |  |
| --- | --- | --- | --- | --- | --- | --- | --- | --- |
|  |  |  |  |  |  |  |  |  |
|  | Keratoconus | Healthy controls | *p* value (two-sided Mann-Whitney-U Test) | Asymmetric keratoconus | | Healthy controls | *p* value (two-sided Mann-Whitney-U Test) |  |
|  |  |  |  | Eyes with tomographically significant keratoconus | Tomographically normal eyes |  |  |  |
| Number of patients (n) | 54 | 20 | n/a | 21 | | 21 | n/a |  |
| Median (IQR) age (years) | 30 (14) | 24 (9) | 0.024 | 29 (11) | | 30 (9) | 0.950 |  |
| Sex (female/male) | 16/38 | 12/8 | n/a | 2/19 | | 10/11 | n/a |  |
| Number of eyes (n) | 86 | 40 | n/a | 21 | 21 | 21 | n/a |  |
| Right/left eyes | 47/39 | 20/20 | n/a | 8/13 | 13/8 | 10/11 | n/a |  |
| Median (IQR) BAD-D value^+^ | 6.4 (4.8) | 1.0 (0.8) | <0.001 | 6.6 (4.7) | 1.1 (0.6) | 0.9 (0.5) | <0.001^1)^; 0.107^2)^ |  |
| Median (IQR) ARC value^+^ (mm) | 7.2 (0.8) | 7.8 (0.4) | <0.001 | 7.3 (0.5) | 7.8 (0.3) | 7.9 (0.4) | <0.001^1)^; 0.199^2)^ |  |
| Median (IQR) PRC value^+^ (mm) | 5.4 (0.9) | 6.3 (0.4) | <0.001 | 5.5 (0.8) | 6.3 (0.4) | 6.4 (0.4) | <0.001^1)^; 0.242^2)^ |  |
| Median (IQR) thinnest pachymetry value^+^ (µm) | 479.0 (55.0) | 550.5 (29.0) | <0.001 | 504.0 (66.0) | 536.0 (34.0) | 552.0 (32.0) | <0.001^1)^; 0.099^2)^ |  |
| Median (IQR) Kmax value of anterior surface^+^ (D) | 50.8 (7.1) | 44.8 (2.6) | <0.001 | 50.2 (4.8) | 44.0 (1.4) | 43.5 (2.4) | <0.001^1)^; 0.222^2)^ |  |
| Median (IQR) mean simulated keratometry value of anterior surface* (D) | 45.3 (3.5) | 43.7 (2.38) | <0.001 | 45.2 (1.5) | 43.3 (1.5) | 42.8 (2.1) | <0.001^1)^; 0.333^2)^ |  |
| Median (IQR) astigmatism value of anterior surface* (D) | 2.6 (2.7) | 1.0 (1.0) | <0.001 | 3.2 (2.9) | 0.7 (1.0) | 0.9 (0.5) | <0.001^1)^; 0.850^2)^ |  |
| Median (IQR) mean ET (µm) | 50.0 (4.0) | 49.0 (4.0) | 0.137 | 50.7 (3) | 50.7 (2.3) | 51.0 (3.8) | 0.696^1)^; 0.870^2)^ |  |
| Median (IQR) SD of mean ET (µm) | 4.0 (2.0) | 2.0 (0) | <0.001 | 4.3 (2.3) | 2.0 (0.8) | 2.0 (0.7) | <0.001^1)^; 0.191^2)^ |  |
| Median (IQR) minimum ET (µm) | 38.0 (7.0) | 42.0 (6.0) | <0.001 | 39.0 (6.7) | 44.0 (3.5) | 44.0 (5.2) | <0.001^1)^; 0.734^2)^ |  |
| Median (IQR) maximum ET (µm) | 60.5 (6.0) | 54.5 (3.0) | <0.001 | 61.3 (5.3) | 56.3 (3.5) | 56.7 (4.0) | <0.001^1)^; 0.267^2)^ |  |
| Median (IQR) mean inferior – superior ET (µm) | 0 (3.0) | 2.0 (2.0) | <0.001 | 0.3 (3.2) | 1.7 (2.3) | 2.0 (1.5) | <0.001^1)^; 0.479^2)^ |  |

ARC – anterior average radius of curvature in a 3-mm-zone centered on the thinnest point of the cornea; BAD-D – Belin/Ambrósio Enhanced Ectasia total deviation index; D – diopters; ET – epithelial thickness; IQR – interquartile range; n/a – not applicable; PRC – posterior average radius of curvature in a 3-mm-zone centered on the thinnest point of the cornea; SD – standard deviation.
