# Supplementary material for: Inter-zonal epithelial thickness differences for early keratoconus detection using optical coherence tomography
Source: Eye (Lond). 2024 Jul 13;38(15):2968–75. doi: 10.1038/s41433-024-03199-7 (PMC11461491; doi:10.1038/s41433-024-03199-7)
Supplement: Supplementary file 2 — Supplementary Table 2 [file 41433_2024_3199_MOESM2_ESM.docx]

**Supplementary Table 2.** The values of the area under the receiver operating characteristic curve for the zonal epithelial thickness and the inter-zonal epithelial thickness differences of each analyzed zone, along with the *p* values for the comparison between keratoconus and healthy eyes in the parameter-development dataset.

| **Analyzed zone** | **Zonal epithelial thickness area under the ROC curve (95 % confidence interval)^a^** | **Inter-zonal epithelial thickness differences area under the ROC curve (95 % confidence interval)** | ***p* value^b^ for zonal epithelial thickness** | ***p* value^b^ for inter-zonal epithelial thickness differences** |
| --- | --- | --- | --- | --- |
| pS | 0.335 (0.235 - 0.435) | 0.644 (0.541 - 0.747) | 0.004 | 0.011 |
| pNSS | 0.362 (0.258 - 0.466) | 0.632 (0.527 - 0.737) | 0.018 | 0.02 |
| pNS | 0.405 (0.298 - 0.511) | 0.665 (0.563 - 0.766) | 0.13 | 0.003 |
| pNNS | 0.420 (0.311 - 0.528) | 0.659 (0.559 - 0.758) | 0.171 | 0.003 |
| pN | 0.445 (0.340 - 0.549) | 0.780 (0.696 - 0.864) | 0.401 | <0.001 |
| pNNI | 0.423 (0.323 - 0.524) | 0.798 (0.718 - 0.879) | 0.243 | <0.001 |
| pNI | 0.405 (0.305 - 0.505) | 0.823 (0.745 - 0.901) | 0.119 | <0.001 |
| pNII | 0.340 (0.243 - 0.437) | 0.851 (0.780 - 0.923) | 0.008 | <0.001 |
| pI | 0.306 (0.212 - 0.401) | 0.840 (0.765 - 0.915) | 0.001 | <0.001 |
| pTII | 0.346 (0.248 - 0.444) | 0.901 (0.848 - 0.955) | 0.008 | <0.001 |
| pTI | 0.462 (0.359 - 0.565) | 0.933 (0.889 - 0.976) | 0.534 | <0.001 |
| pTTI | 0.480 (0.374 - 0.586) | 0.921 (0.875 - 0.968) | 0.836 | <0.001 |
| pT | 0.383 (0.277 - 0.488) | 0.851 (0.783 - 0.920) | 0.035 | <0.001 |
| pTTS | 0.334 (0.236 - 0.433) | 0.761 (0.675 - 0.847) | 0.003 | <0.001 |
| pTS | 0.336 (0.239 - 0.432) | 0.730 (0.638 - 0.822) | 0.003 | <0.001 |
| pTSS | 0.312 (0.214 - 0.409) | 0.684 (0.582 - 0.786) | <0.001 | 0.001 |
| iS | 0.281 (0.188 - 0.374) | 0.726 (0.633 - 0.820) | <0.001 | <0.001 |
| iNSS | 0.323 (0.224 - 0.421) | 0.747 (0.658 - 0.836) | 0.002 | <0.001 |
| iNS | 0.329 (0.229 - 0.429) | 0.729 (0.639 - 0.819) | 0.003 | <0.001 |
| iNNS | 0.343 (0.243 - 0.443) | 0.835 (0.763 - 0.907) | 0.006 | <0.001 |
| iN | 0.362 (0.260 - 0.463) | 0.813 (0.737 - 0.888) | 0.014 | <0.001 |
| iNNI | 0.367 (0.267 - 0.468) | 0.892 (0.833 - 0.950) | 0.023 | <0.001 |
| iNI | 0.363 (0.262 - 0.463) | 0.880 (0.819 - 0.941) | 0.022 | <0.001 |
| iNII | 0.362 (0.260 - 0.465) | 0.918 (0.867 - 0.969) | 0.022 | <0.001 |
| iI | 0.443 (0.338 - 0.549) | 0.939 (0.898 - 0.981) | 0.311 | <0.001 |
| iTII | 0.593 (0.490 - 0.695) | 0.958 (0.927 - 0.989) | 0.093 | <0.001 |
| iTI | 0.701 (0.605 - 0.797) | 0.944 (0.905 - 0.982) | <0.001 | <0.001 |
| iTTI | 0.708 (0.612 - 0.803) | 0.951 (0.914 - 0.987) | <0.001 | <0.001 |
| iT | 0.501 (0.394 - 0.607) | 0.906 (0.855 - 0.958) | 0.933 | <0.001 |
| iTTS | 0.365 (0.265 - 0.465) | 0.865 (0.800 - 0.930) | 0.018 | <0.001 |
| iTS | 0.304 (0.209 - 0.400) | 0.770 (0.685 - 0.855) | <0.001 | <0.001 |
| iTSS | 0.276 (0.186 - 0.367) | 0.752 (0.662 - 0.841) | <0.001 | <0.001 |
| cS | 0.296 (0.200 - 0.392) | 0.818 (0.744 - 0.892) | <0.001 | <0.001 |
| cNS | 0.296 (0.201 - 0.391) | 0.865 (0.802 - 0.928) | <0.001 | <0.001 |
| cN | 0.374 (0.273 - 0.475) | 0.907 (0.851 - 0.962) | 0.027 | <0.001 |
| cNI | 0.584 (0.481 - 0.688) | 0.947 (0.909 - 0.986) | 0.102 | <0.001 |
| cI | 0.778 (0.692 - 0.864) | 0.974 (0.951 - 0.997) | <0.001 | <0.001 |
| cTI | 0.848 (0.780 - 0.916) | 0.970 (0.940 - 0.999) | <0.001 | <0.001 |
| cT | 0.663 (0.565 - 0.761) | 0.952 (0.916 - 0.988) | 0.003 | <0.001 |
| cTS | 0.403 (0.300 - 0.507) | 0.855 (0.788 - 0.923) | 0.089 | <0.001 |
| C | 0.601 (0.498 - 0.704) | 0.921 (0.872 - 0.969) | 0.071 | <0.001 |

a - for the purpose of the receiver operating characteristic analysis, lower zonal epithelial thickness values were considered to be indicative for keratoconus. The significance level after Bonferroni‘s correction for 82 comparisons was 0.001. ROC – receiver operating characteristic. b - two-sided Mann-Whitney-U test for keratoconus eyes vs. healthy eyes.
