# Supplementary figures and images for: Inter-zonal epithelial thickness differences for early keratoconus detection using optical coherence tomography
Source: Eye (Lond). 2024 Jul 13;38(15):2968–75. doi: 10.1038/s41433-024-03199-7 (PMC11461491; doi:10.1038/s41433-024-03199-7)

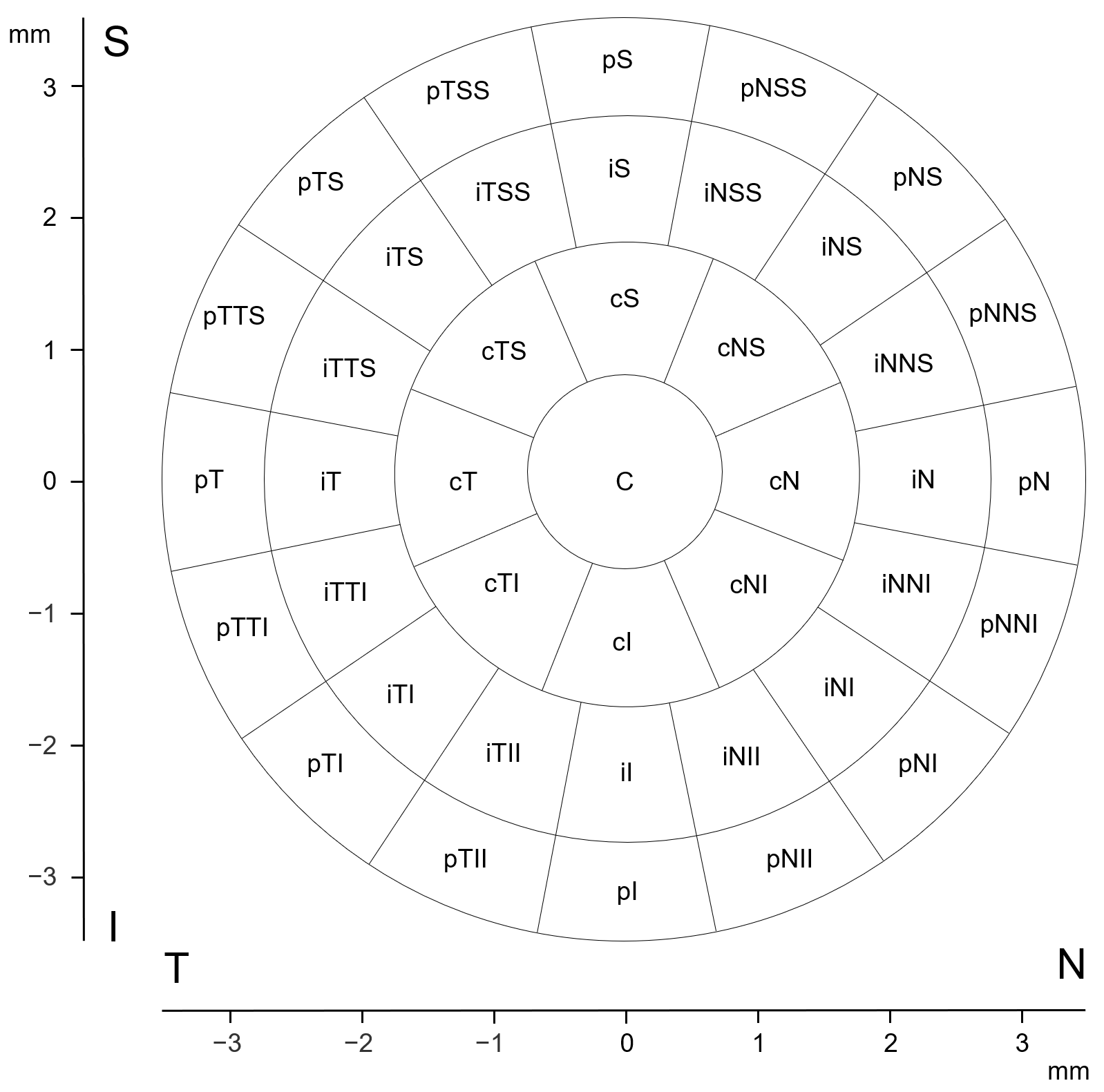

Supplement: Supplementary file 3 — Supplementary Figure 1 [file 41433_2024_3199_MOESM3_ESM.tif]

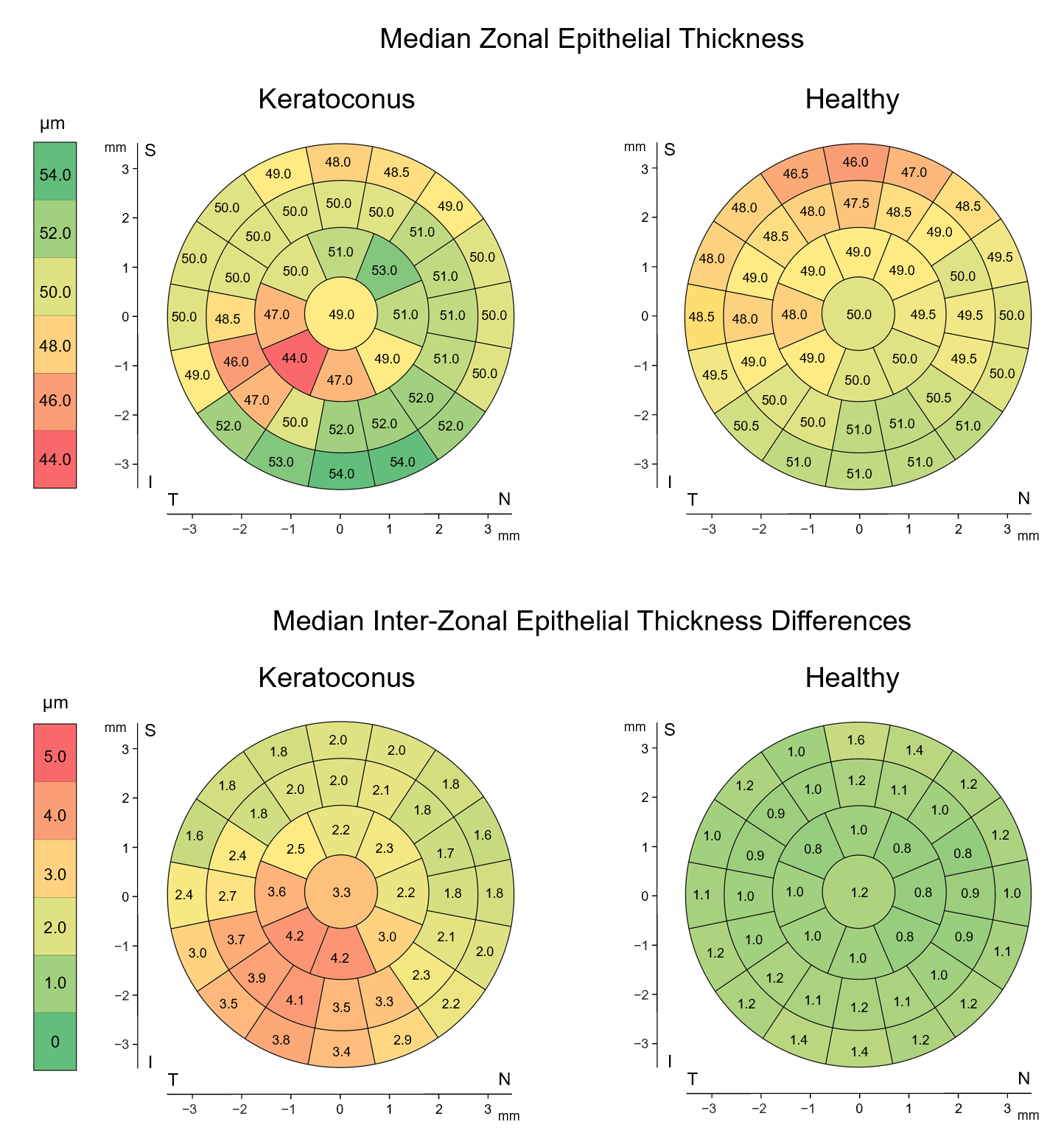

Supplement: Supplementary file 4 — Supplementary Figure 2 [file 41433_2024_3199_MOESM4_ESM.tif]

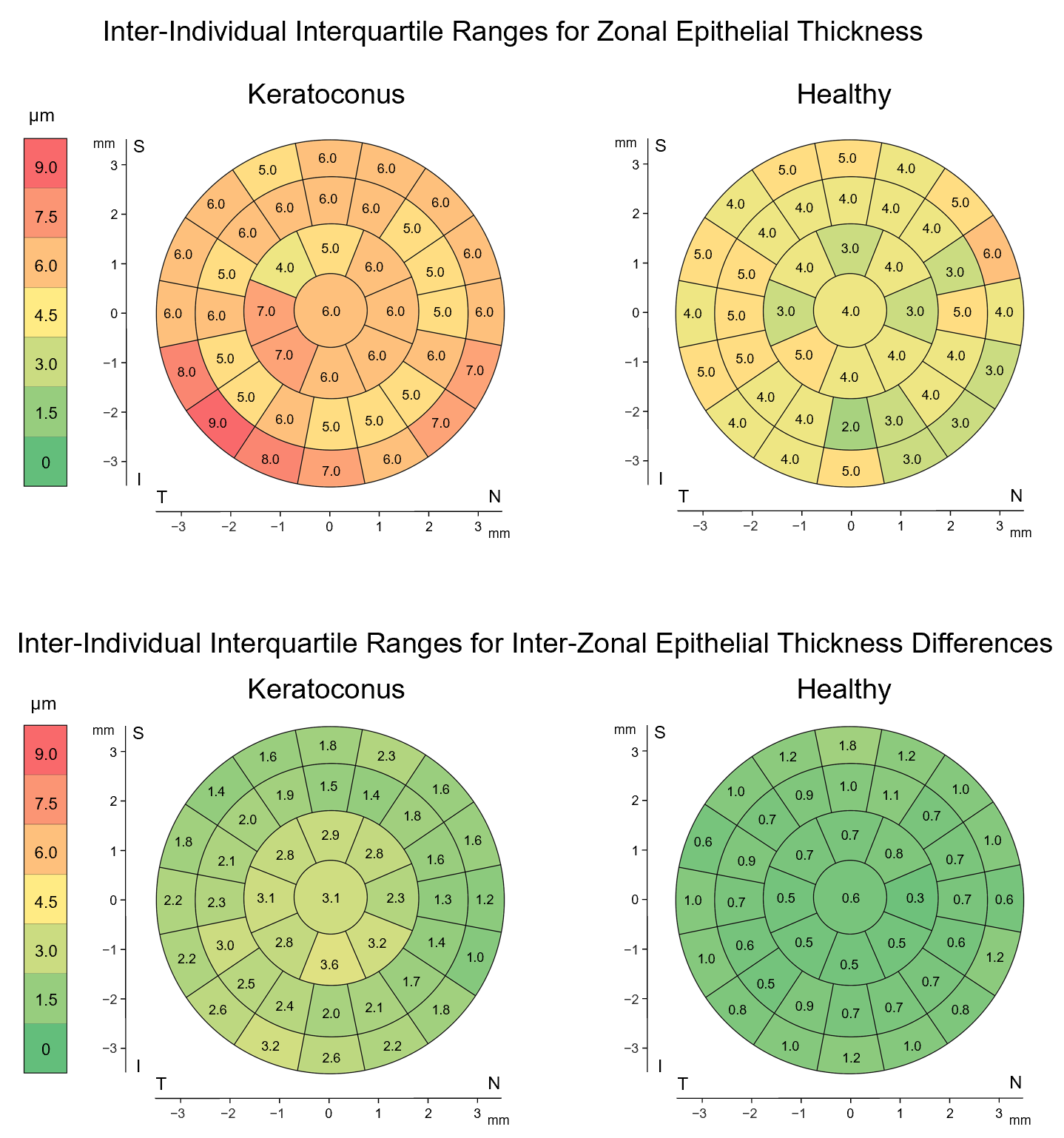

Supplement: Supplementary file 5 — Supplementary Figure 3 [file 41433_2024_3199_MOESM5_ESM.tif]

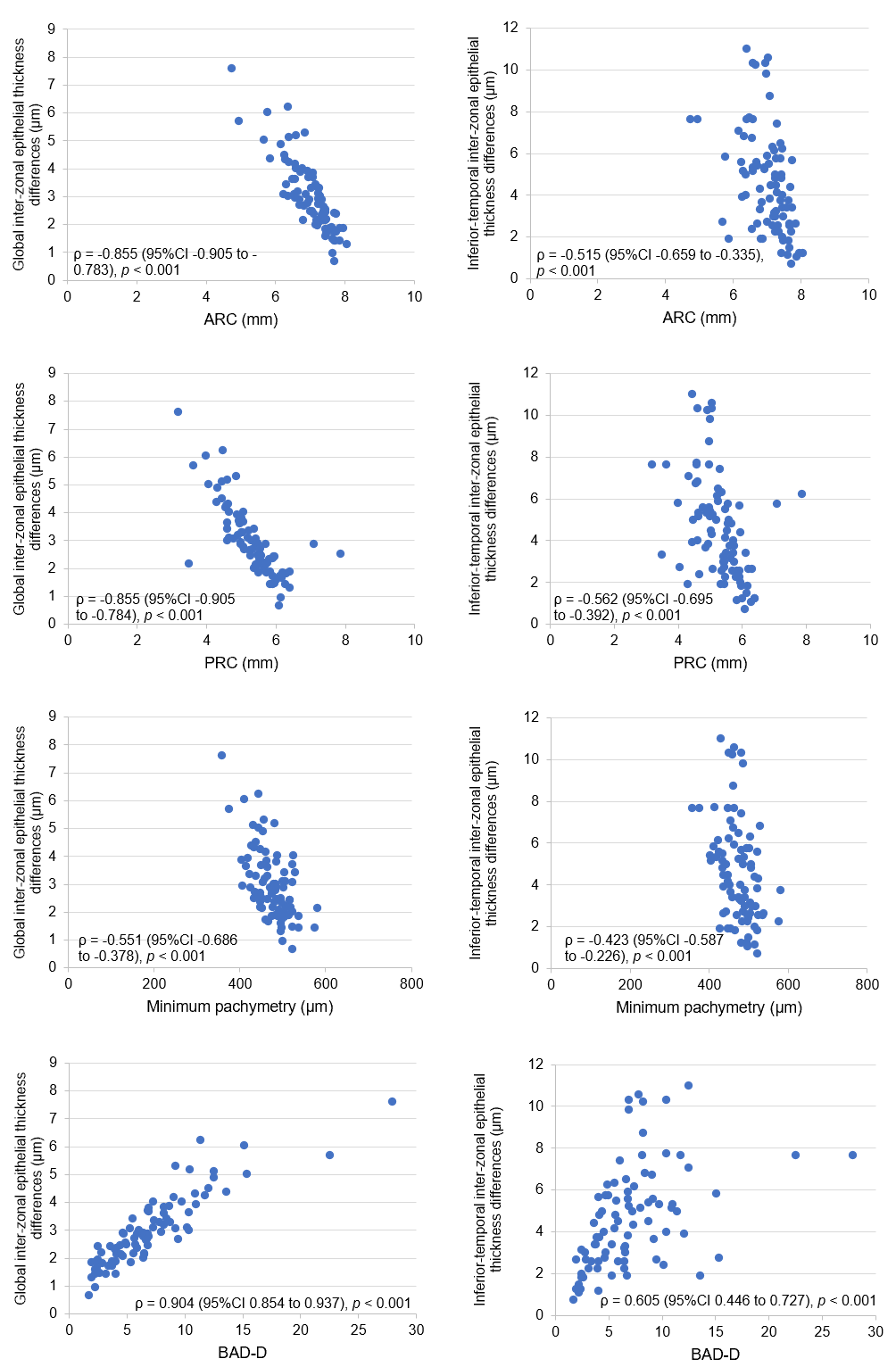

Supplement: Supplementary file 6 — Supplementary Figure 4 [file 41433_2024_3199_MOESM6_ESM.tif]

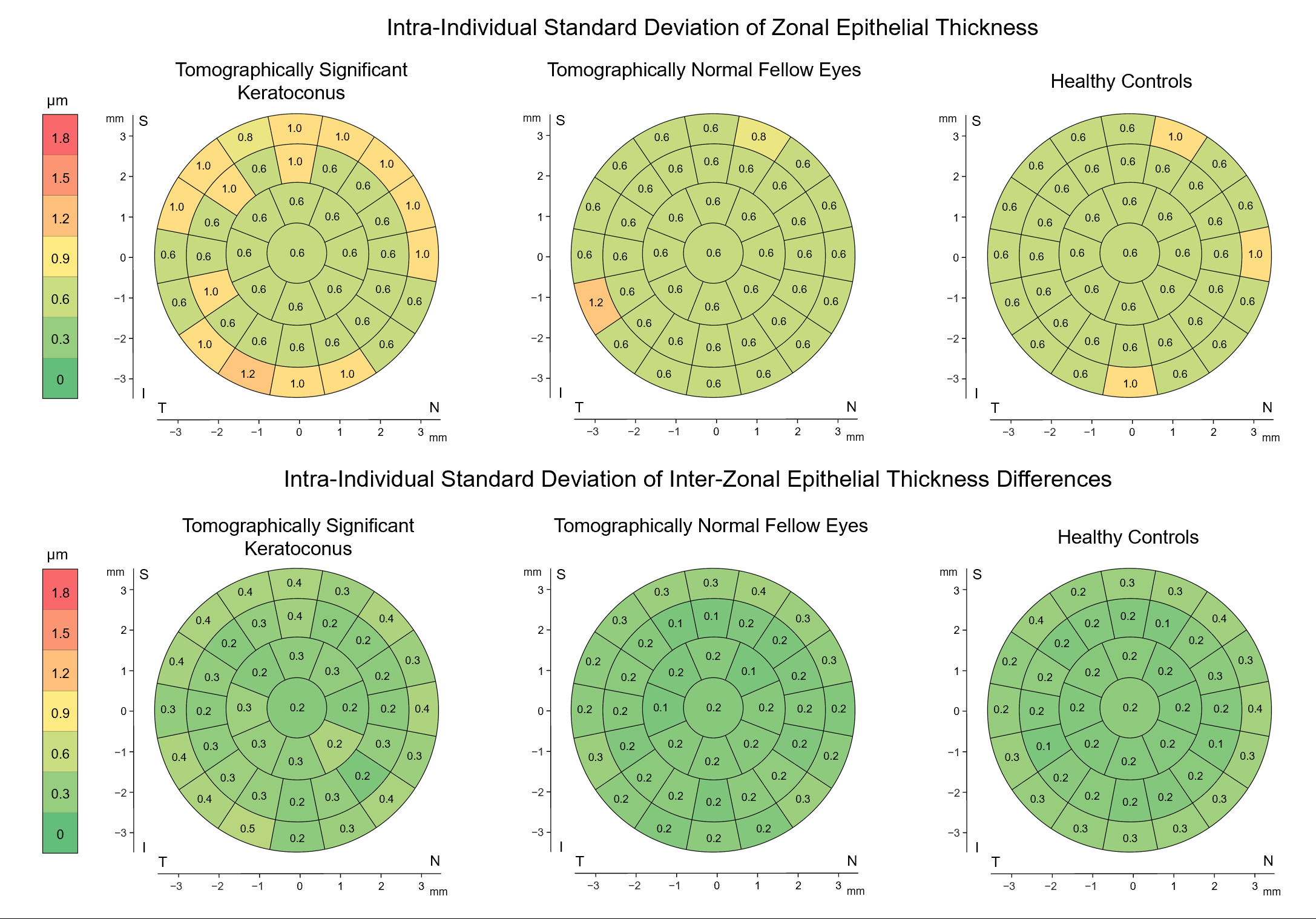

Supplement: Supplementary file 7 — Supplementary Figure 5 [file 41433_2024_3199_MOESM7_ESM.tif]
