# Supplementary material for: Inter-zonal epithelial thickness differences for early keratoconus detection using optical coherence tomography
Source: Eye (Lond). 2024 Jul 13;38(15):2968–75. doi: 10.1038/s41433-024-03199-7 (PMC11461491; doi:10.1038/s41433-024-03199-7)
Supplement: Supplementary file 8 — Supplementary materials [file 41433_2024_3199_MOESM8_ESM.docx]

**Supplementary materials**

**Supplementary Figure 1**. The zones of the epithelial thickness map with an overall diameter of 7mm. I–inferior; N–nasal; S–superior; T–temporal; C–central; c–paracentral; i–intermediate; p–peripheral.

**Supplementary Figure 2**. Median zonal epithelial thickness and median inter-zonal epithelial thickness differences of the eyes included in the parameter-development dataset. All values in the maps are in µm. I–inferior; N–nasal; S–superior; T–temporal.

**Supplementary Figure 3**. Inter-individual variation of zonal epithelial thickness values and of inter-zonal epithelial thickness differences, expressed as interquartile ranges in keratoconus and healthy eyes in the parameter-development dataset. All values in the maps are in µm. I–inferior; N–nasal; S–superior; T–temporal.

**Supplementary Figure 4.** Scatter plots with the Spearman correlation coefficients (ρ) and *p* values for the correlation between the tomographical and epithelial thickness parameters in keratoconus eyes included in the parameter-development dataset (n=86). ARC–anterior average radius of curvature in a 3-mm-zone centred on the thinnest point of the cornea; BAD-D–Belin/Ambrósio Enhanced Ectasia total deviation index; PRC–posterior average radius of curvature in a 3-mm-zone centred on the thinnest point of the cornea.

**Supplementary Figure 5**. Median intra-subject standard deviation values of the three measurements of the eyes included in the parameter-validation dataset, indicating the repeatability of the parameters analysed. All values in the maps are in µm. I–inferior; N–nasal; S–superior; T–temporal.

**Supplementary Table 1.** Characteristics of patients and eyes included in the study.

**Supplementary Table 2.** The values of the area under the receiver operating characteristic curve for the zonal epithelial thickness and the inter-zonal epithelial thickness differences of each analyzed zone, along with the *p* values for the comparison between keratoconus and healthy eyes in the parameter-development dataset.
